# Evaluation of the *in vivo* and *in vitro* safety profile of *Cuscuta epithymum* ethanolic extract

**DOI:** 10.22038/AJP.2021.18529

**Published:** 2021

**Authors:** Mohammad Reza Abedini, Samaneh Paki, Mahtab Mohammadifard, Mohsen Foadoddini, Khadijeh Vazifeshenas-Darmiyan, Mehran Hosseini

**Affiliations:** 1 *Cellular and Molecular Research Center, Department of Pharmacology, Birjand University of Medical Sciences, Birjand, Iran*; 2 *Student Research Committee, Birjand University of Medical Sciences, Birjand, Iran*; 3 *Department of Pathology, Faculty of Medicine, Birjand University of Medical Sciences, Birjand, Iran*; 4 *Department of Physiology, Faculty of Medicine, Birjand University of Medical Sciences, Birjand, Iran*; 5 *Cellular and Molecular Research Center, Department of Biochemistry, Birjand University of Medical Sciences, Birjand, Iran*; 6 *Cellular and Molecular Research Center, Department of Anatomical Sciences, Birjand University of Medical Sciences, Birjand, Iran*

**Keywords:** Acute toxicity, Cuscuta, Cytotoxicity, Dodder, Sub-acute toxicity

## Abstract

**Objective:**

*Cuscuta epithymum* (CE) is one of the most popular medicinal plants in the world. However, detailed information about its toxicity is not available. Hence, this study aimed to evaluate the safety profile of CE ethanolic extract *in vitro* and *in vivo*.

**Materials and Methods:**

The extract's *in vitro* toxicity profile was investigated on normal fibroblast and cervical cancer cells by cytotoxicity test. In the next step, acute oral and intraperitoneal (i.p.) toxicity of the CE extract was evaluated in Wistar rats and BALB/c mice, respectively. Sub-acute oral toxicity was also examined by administering repeated oral doses of the CE extract (50, 200, and 500 mg/kg) to Wistar rats for 28 days.

**Results:**

The CE extract exhibited a significant cytotoxicity on both normal (IC_50_ 0.82 mg/ml, p<0.001) and cancer cells (IC_50_ 1.42 mg/ml, p<0.001). Acute oral administration of a single dose of CE extract (175-5000 mg/kg) did not cause mortality; however, its i.p. administration caused mortality at doses greater than 75 mg/kg (i.p. LD_50_ 154.8 mg/kg). In the sub-acute toxicity test, no significant effects in terms of weight change, organ weights, blood chemistry, or kidney pathology were observed. However, at 200 and 500 mg/kg doses, the CE extract significantly increased liver pathological scores compared to the control group (p<0.05 and p<0.01, respectively).

**Conclusion:**

CE exhibited toxicities in i.p. acute and repeated oral dose administrations. It showed identical cytotoxicity against normal and cancer cells. This herb must be prescribed cautiously by traditional medicine practitioners.

## Introduction

Nowadays, the usage of medicinal plants is increasing around the world (Caporale et al., 2020 ▶). Medicinal plants are generally considered safe due to their natural origin and cultural acceptability. This assumption may lead to their indiscriminate use. Nevertheless, several experimental studies and clinical reports have found that medicinal plants might potentially have side effects like synthetic drugs (Farzaei et al., 2020 ▶). *Cuscuta epithymum* (dodder) is a parasitic and not photosynthetically active plant assigned to the Convolvulaceae family. It has slender stems with small leaves appearing in different colors like yellow, red, purple, and pink (Costea and Tardif, 2006 ▶). The phytochemical properties of *C. epithymum* (CE) vary based on its host. CE has several secondary metabolites such as saponins, glycosides, tannins, steroids, kaempferol, and quercetin (Chabra et al., 2019 ▶). Traditionally, CE has been used for the treatment of insanity (Iran), diabetes (Morocco), burn injuries (India), psychometric disorders (India), liver disorders (India), vision improvement (Greece), and rheumatism (China) (Kong and Chen, 1996 ▶; Jouad et al., 2001 ▶; Rout et al., 2013 ▶; Sharma et al., 2014 ▶; Shah et al., 2015 ▶). According to the results of an ethnobotanical study conducted in Iran, CE (in Persian: *Aftimun*) was reported as one of the top-selling products in traditional herbal medicine markets (Amiri and Joharchi, 2013 ▶). Previous experimental studies have found that CE showed several pharmacological activities, including antioxidant, antifungal, antibacterial, hepato-protective, anticonvulsant, cytotoxic, and sedative-hypnotic properties (Mehrabani et al., 2007 ▶; Ganapaty et al., 2013 ▶; Jafarian et al., 2014 ▶; Sudam et al., 2017 ▶; Chabra et al., 2019 ▶; Forouzanfar et al., 2020 ▶). Given the above evidence, the use of CE for the clinical management of various diseases is expected to increase. For example, a recent clinical study has been investigated CE capsule (500 mg) effects in patients with schizophrenia (Parvizi et al., 2019 ▶).

Regardless of the pharmacological benefits of CE, detailed information about its toxicological profile is not available. Hence, the present study was performed to evaluate *in vitro* and *in vivo* toxicological profile of CE ethanolic extract.

## Materials and Methods

**Chemicals**

Dulbecco’s modified Eagle’s medium (DMEM) and fetal bovine serum (FBS) were purchased from Biosera, France. Trypsin was procured from Gibco Company, Canada. Phosphate-buffered saline (PBS), 3-(4, 5-dimethylthiazol-2-yl)-2, 5-diphenyltetrazolium bromide (MTT), and dimethyl sulfoxide were purchased from Sigma-Aldrich, USA.

**Extract preparation**

The whole plant of CE was harvested from the agriculture research farm at the agriculture faculty of the University of Birjand, Birjand, Iran. A voucher specimen (code: 643) was deposited to the herbarium of the University of Birjand, faculty of agriculture, Birjand, Iran. 

The whole plant (seedpods as well as steams) was air-dried at room temperature. It was powdered using an electric miller and macerated in 80% ethanol (1:10 w/v) for 48 hr at room temperature. The resulting mixture was passed through filter papers (Blue Ribbon, Grade 589, Germany) and concentrated under a vacuum evaporator (Wiggens, Italy) at 45°C. The resulting residue was transferred to 120-mm Petri dishes (10 ml per dish) and allowed to dry at 45°C (Ghiravani et al., 2016 ▶). The yield of extraction was around 17.5%. 

**Cytotoxicity assay**

Human primary dermal normal fibroblast cells (HDNF) (C654, Pasteur Institute of Iran, Iran) and cisplatin-resistant human cervical cancer cells (C13*) (kindly supplied by Dr. Benjamin K. Tsang’s Laboratory, University of Ottawa, Canada) were used for cytotoxicity assay. Cells were grown in the DMEM supplemented with 10% FBS at 37°C in 5% CO_2_ until 80% confluence reached (Abedini et al., 2014 ▶). Subsequently, cells were trypsinized and transferred into a 96-well plate and incubated (12 hr) at 37°C in 5% CO_2_. Next, the medium was replaced with PBS overnight for cell starving, and then cells were exposed to ethanolic extract of CE at a two-fold concentration (0.15-5 mg/ml) for 24 hr at 37°C in 5% CO_2_ (Fiume et al., 2014). The control wells were maintained with PBS.

In order to determine cell viability, the MTT dye was used. The cells were rinsed with PBS and incubated with 0.5 mg/ml MTT diluted in complete DMEM for 4 hr. Then, supernatants were removed, dimethyl sulfoxide (150 µl) was added to each well, and the plate was incubated for 10 min. The absorbance was read at 570 nm using a 96-well ELISA plate reader (BioTek, Vermont, USA). Five replicates for each extract concentration were performed. The percentage of cell viability was calculated as follows: 

Percentage of cell viability = (A_ treatment_ – A _blank_)/(A _control_ – A _blank_) × 100 (where A = absorbance) (Hoshyar et al., 2015 ▶).

***In vivo*** **toxicity assessment**

**Animals**

All procedures involving animals were performed in accordance with the national guides for the care and use of Laboratory Animals in Scientific Affairs provided by the Iranian Ministry of Health and Medical Education (2020). The guideline complies with the ARRIVE guidelines (Percie du Sert et al., 2020 ▶). The animal experiments were approved by the Birjand University of Medical Sciences ethics committee (permit code: Ir.bums.REC.1398.052).

*In vivo* experiments were performed in both BALB/c mice (male, 8-week old), and Wistar rats (both sexes, 8-week old). Animals were housed under standard conditions (24±2ºC, 30-35% humidity and a 12 hr light/dark cycle) and fed with a standard diet (Behparvar Co, Iran) and tap water *ad libitum*. 

In the present study, three main types of experiments were used to assess the CE extract toxicity: 1-Acute intraperitoneal (i.p.) toxicity assessment upon single administration of three doses (75, 150, and 300 mg/kg) to male BALB/c mice. 2-Acute oral toxicity assessment upon single administration of four doses (175, 550, 1750, and 5000 mg/kg) to female Wistar rats. 3- Sub-acute oral toxicity assessment upon repeated (28 days) administrations of three doses (50, 200, and 500 mg/kg) to male and female Wistar rats.

**Acute i.p. toxicity experiment**

A two-fold concentration gradient test was performed to determine the *in vivo* i.p. LD_50 _(median lethal dose) value for the CE extract. Accordingly, three different doses (75, 150, and 300 mg/kg) were tested. For each dose, five mice were injected (i.p.) once and monitored for five days (Nagarajan et al., 2019 ▶; Askari et al., 2021 ▶). The minimum i.p. dose of CE extract (75 mg/kg) was selected according to previous works (Mehrabani et al., 2007 ▶; Forouzanfar et al., 2020 ▶).

**Acute oral toxicity experiment**

An acute oral toxicity test for calculating oral LD_50_ was performed according to the method described in OECD Test Guideline 425 (up and down procedure). In this method, animal's death/survival determines dose progression/decrease. Based on the guideline recommendation, four different doses (175, 550, 1750, and 5000 mg/kg) of the CE extract were tested in female Wistar rats. In general, female rats are assumed to be more sensitive than males to the acute toxic effects of substances (Lipnick et al., 1995 ▶). In brief, a female Wistar rat received a single dose of 175 mg/kg of the CE extract by gavage and monitored for mortality and any signs of abnormality periodically during the first 30 min, then for 4 hr and finally, once-daily for 14 days. Upon survival of the treated rat after 48 hr, four additional rats were administered with the same dose. The same procedure was performed for the other proposed doses (550, 1750, and 5000 mg/kg). As there was no information regarding the oral LD_50_ dose of the CE extract, the starting dose of 175 mg/kg and the default dose progression factor of 3.2 were selected based on the OECD Test Guideline 425 recommendation (Rispin et al., 2002 ▶). Simultaneously, a control group was assigned that only received vehicle solution (saline). Parameters including body weight change, water intake, and food consumption were documented.

**Sub-acute oral toxicity experiment**

A sub-acute oral toxicity assay was conducted according to the OECD Test Guidelines 407. On that account, Wistar rats were randomly divided into four equal groups (a control and three extract groups) consisting of 10 rats each (5 female and 5 male). The CE extract at doses of 50 (CE50), 200 (CE200) and 500 mg/kg (CE500) were administered orally in three experimental groups for 28 consecutive days (Hassanzadeh-Taheri et al., 2018b ▶). Instead, the control group only received vehicle solution (saline) in the same volume of the experimental groups. Two to four-fold intervals are frequently optimal for setting the descending dose levels in the sub-acute oral toxicity assay (Sutrisni et al., 2019 ▶). Based on this concept and the results of the i.p. oral toxicity test, dose levels were determined as follows: low dose group (50 mg/kg), medium-dose group (200 mg/kg), and high dose group (500 mg/kg).

At the end of the study, rats were fasted overnight, weighed and anesthetized with ketamine-xylazine (65:10 mg/kg i.p) (Hassanzadeh-Taheri et al., 2016 ▶). Blood samples were drawn via cardiac puncture to determine the levels of creatinine (Cr), urea, aspartate transaminase (AST), and alanine transaminase (ALT). Immediately after blood collection, vital organs including the lung, heart, liver, kidney, spleen, testis, or ovary were dissected out and weighed. 

Samples from the liver and kidney (left kidney) were fixed in 4% paraformaldehyde solution for histological evaluation. Tissue specimens were routinely processed and embedded in paraffin wax, sectioned (5-µm thickness), and stained with hematoxylin and eosin. Three random slides from each sample were examined under a light microscope (Euromex-CMEX-10, Netherlands). Pathological lesions were quantified according to a scoring checklist in which pathological features like hemorrhage, infiltration, congestion, and degeneration were scored (Hassanzadeh-Taheri et al., 2018b ▶; Moodi et al., 2020 ▶). Accordingly, the score of each item was recorded as one of the following five grades: 1 (normal), 2 (slight injury involving up to 25% of the microscopic field), 3 (moderate injury involving 25-50% of the microscopic field), 4 (severe damage involving 50-75% of the microscopic field and 5 (very severe damage involving more than 75% of the microscopic field). Each item was scored from 1 to 5, as described above. The final score was calculated based on the sum of all scored samples of each group.

**Statistical analysis**

Data were analyzed using the statistical software IBM SPSS version 22. Values are presented as mean±standard deviation (SD). The normality of data was checked using the Shapiro-Wilk normality test. Statistical comparisons were performed using one-way analysis of variance (ANOVA), and *post hoc* analysis was done using Dunnett's test. The Kruskal-Wallis test was used to compare the pathological scores among the studied groups. A p<0.05 was considered to indicate a statistically significant difference.

## Results

***In vitro*** **toxicity results**

**Figure 1:**
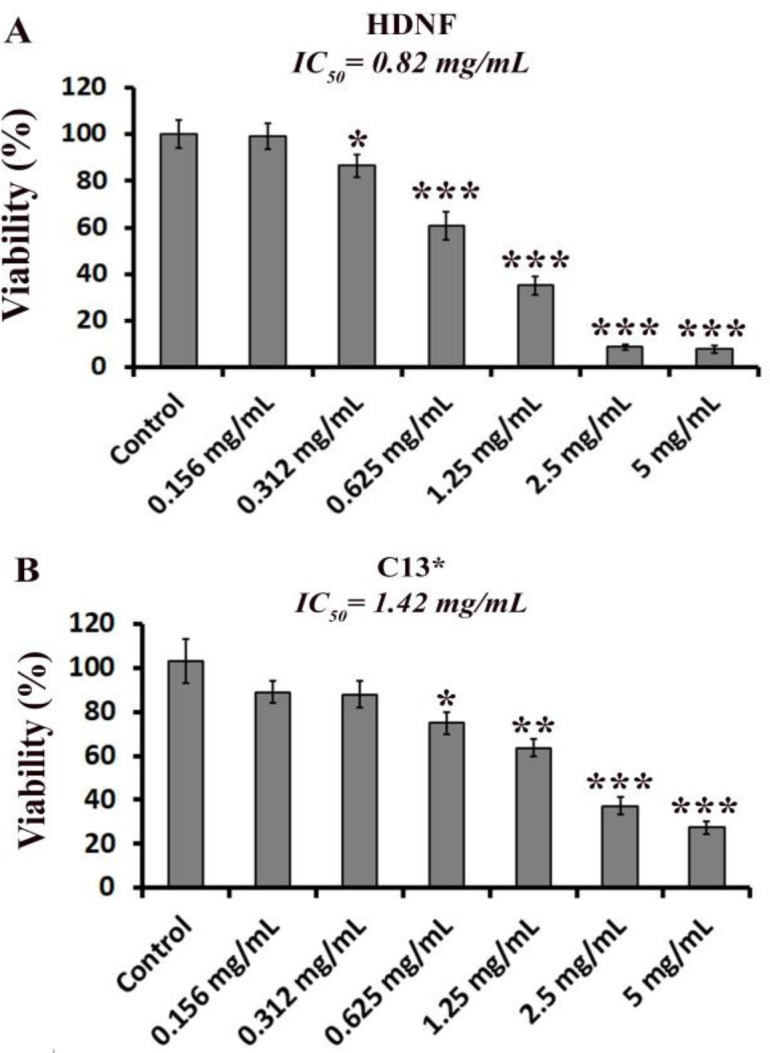
Cytotoxicity of ethanolic extract of *Cuscuta epithymum* (0.156-5 mg/ml) on human dermal normal fibroblast cells (HDNF) (A) and human cisplatin-resistant cervical cancer cells (C13*) (B). Differences are represented as *p<0.05, **p<0.01 and ***p<0.001 compared to the control group.

*In vitro* cytotoxicity evaluation of the CE ethanolic extract in HDNF and C13* cells was performed using the MTT method. The results of the cytotoxicity assay are shown in Figure 1. The half-maximal inhibitory concentration (IC_50_) values were calculated using a linear regression equation (Askari et al., 2021 ▶). The 24 hr IC_50_ values obtained for the CE extract in HDNF and C13* cells were 0.82 and 1.42 mg/ml, respectively.

**Results of acute i.p. toxicity**

**Figure 2:**
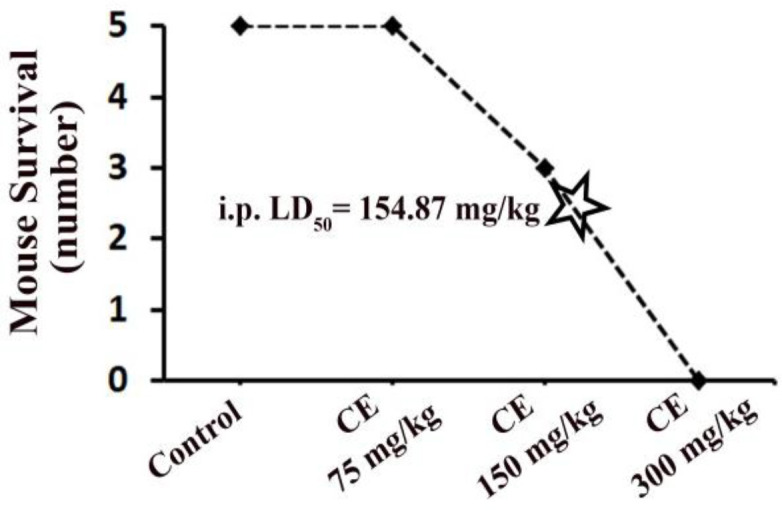
Determination of* in vivo* intraperitoneal LD_50_ (median lethal dose) for ethanolic extract of *Cuscuta epithymum* in BALB/c mice (n=5 mice per dose)

Based on the acute i.p. toxicity assay results, the i.p. LD_50_ value for the CE extract was estimated 154.87 mg/kg. The maximum sub-lethal i.p. dose of CE extract was 75 mg/kg (Figure 2).

**Results of acute oral toxicity**

Single oral treatment of female rats with the CE extract at 175- 5000 mg/kg doses did not cause any death. Therefore, the oral LD_50_ value for the CE extract was more than 5000 mg/kg. Behavioral changes including hypo-activity and asthenia were observed 1 hr following CE administration at 1750 and 5000 mg/kg doses and disappeared after 24 hr. There was no significant difference in weight change, food consumption, or water intake among the studied groups 14 days after CE administrations (Table 1). 

**Table 1:** Effects of acute oral administration of ethanolic extract of *Cuscuta epithymum* (CE) in female Wistar rats

	**Groups**					ANOVA p-value	
	**CE doses (mg/kg)**						
	**0**	**175**	**550**	**1750**	**500** **0**		
**Weight change (g)**	10.20±1.09	12.00±2.34	11.6±3.13	11.4±1.67	9.80±5.63		0.77
**Food consumption (g/24hr/rat)**	14.40±2.30	14.00±1.22	13.01±1.00	14.20±1.78	14.8±1.09		0.46
**Water intake (ml/24hr/rat)**	21.6±2.60	23.20±2.38	23.20±1.92	23.00±2.34	23.4±2.30		0.87

**Results of sub-acute oral toxicity**

The results of water intake, food consumption, body weight change, and organ weights are presented in Table 2. These results demonstrated that 28 days of repetitive CE administrations at all doses (50-500 mg/kg) could not statistically affect weight change, organ weights, food consumption or, water intake in female or male rats.

The results of the biochemical study are presented in Table 3. Only a statistical difference was found for blood urea among the studied groups. CE treatment at 500 mg/kg significantly decreased blood urea level in male (p=0.034) and female (p=0.04) rats compared to the control groups. No significant changes were observed in fasting blood glucose, Cr, AST, ALT, or total cholesterol levels among the studied groups (both sexes).

**Table 2:** Effects of sub-acute oral administration (28 days) of ethanolic extract of *Cuscuta epithymum* (CE) on weight change, organ weights, and food and water intake in Wistar rats

	**Groups**				ANOVA p-value	
	**Control**	**CE** **50 mg/kg**	**CE** **200 mg/kg**	**CE** **500 mg/kg**		
Male						
Weight change (g)	11.40±8.93	11.61±8.9	12.4±2.07	13.8±4.01	0.31	
Food consumption (g/24hr/rat)	20.92±1.52	19.40±1.89	17.7±3.48	18.8±3.83	0.42	
Water intake (ml/24hr/rat)	40.4±4.03	41.00±3.46	37.6±1.94	36.00±2.44	0.06	
Heart weight (g)	1.21±0.09	1.21±0.1	1.12±0.16	1.15±0.17	0.67	
Lung weight (g)	1.85±0.13	1.94±0.49	1.68±0.06	1.73±0.09	0.43	
Liver weight (g)	9.21±1.36	8.65±1.21	7.92±0.59	8.76±1.21	0.37	
Kidney weight (g)	1.18±0.12	1.05±0.03	1.05±0.05	1.10±0.07	0.054	
Spleen weight (g)	1.28±0.35	1.41±0.04	1.07±0.21	1.11±0.19	0.11	
Testis weight (g)	1.67±0.12	1.62±0.26	1.71±0.10	1.60±0.23	0.81
Female						
Weight change (g)	14.4±9.83	15.8±8.68	11.40±6.50	14.20±4.08	0.17	
Food consumption (g/24hr)	11.84±1.45	10.80±1.30	10.40±1.14	11.20±1.30	0.37	
Water intake (ml/24hr)	25.40±1.14	22.6±3.2	25.8±1.3	23.00±3.39	0.13	
Heart weight (g)	0.92±0.14	0.97±0.17	0.87±0.07	0.94±0.11	0.68	
Lung weight (g)	1.38±0.11	1.48±0.07	1.32±0.06	1.40±0.16	0.22	
Liver weight (g)	6.15±0.70	6.75±0.26	6.65±1.55	5.95±0.5	0.45	
Kidney weight (g)	0.73±0.01	0.72±0.05	0.73±0.07	0.78±0.09	0.505	
Spleen weight (g)	0.82±0.29	0.63±0.07	0.84±0.18	0.97±0.13	0.075	
Testis weight (g)	0.08±0.01	0.06±0.02	0.08±0.013	0.08±0.011	0.53	

Values are expressed as mean±SD, n=5

**Table 3:** Effects of sub-acute oral administration (28 days) of ethanolic extract of *Cuscuta epithymum* (CE) on biochemical parameters in Wistar rats

	**Groups**				ANOVA p-value
	**Control**	**CE** **50 mg/kg**	**CE** **200 mg/kg**	**CE** **500 mg/kg**	
Male					
Glucose (mg/dl)	99.2±7.69	98.2±5.35	96.20±4.54	101.20±7.62	0.67
Blood Urea (mg/dl)	56.60±6.73	41.00±6.08	46.75±5.73	39.20±4.60*	0.029
Blood Creatinine (mg/dl)	0.84±0.05	0.84±0.05	0.75±0.12	0.82±0.08	0.36
Cholesterol (mg/dl)	65.80±17.06	57.80±10.61	51.75±7.50	65.00±11.91	0.23
AST(U/L)	98.20±7.39	108.20±9.47	98.00±26.69	91.20±15.25	0.08
ALT (U/L)	62.60±2.60	67.60±2.50	58.25±3.09	76.80±18.49	0.061
Female					
Glucose (mg/dl)	95.94±7.10	102.00±3.16	97.00±4.69	93.8±4.02	0.75
Blood Urea (mg/dl)	47.40±7.66	52.00±6.27	45.60±7.564	39.00±3.56*	0.04
Blood Creatinine (mg/dl)	0.86±0.11	0.82±0.05	0.82±0.30	0.82±0.04	0.97
Cholesterol (mg/dl)	60.80±6.79	55.50±3.87	64.80±5.04	58.60±8.34	0.27
AST (U/L)	82.20±9.25	80.00±12.98	90.00±14.62	91.80±9.88	0.37
ALT (U/L)	63.20±9.31	58.75±8.18	71.00±16.44	50.40±8.61	0.07

Values are expressed as mean±SD. AST: aspartate transaminase; ALT: alanine transaminase. Statistical analysis was performed using one-way ANOVA. Differences (Dunnett’s multiple comparison test) are represented as *p<0.05 compared to the control group.

**Histopathological results**

**Figure 3:**
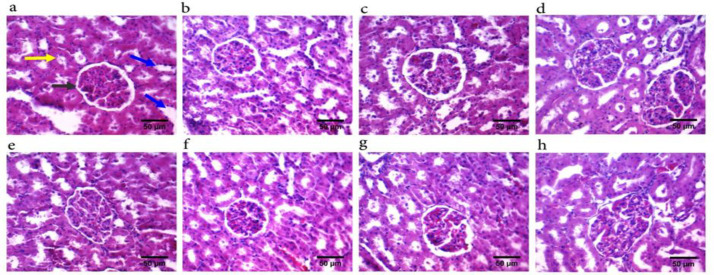
Kidney histopathological results of 28 days of oral administration of *Cuscuta epithymum* (CE) ethanolic extract. Kidney micrographs of male rats treated with saline (a), 50 mg/kg of CE (b), 200 mg/kg of CE (c), and 500 mg/kg of CE (d). Kidney micrographs of female rats treated with saline (e), 50 mg/kg of CE (f), 200 mg/kg of CE (g), and 500 mg/kg of CE (H). Proximal convoluted tubule (yellow arrow), distal convoluted tubule (blue arrow), and glomerulus (black arrow). Hematoxylin and eosin staining, 400X magnification (scale-bar 50µm)

**Figure 4:**
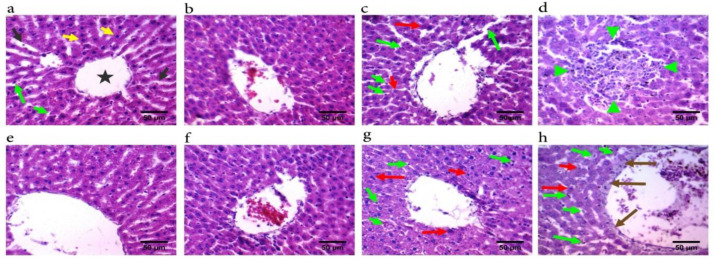
Liver histopathological results of 28 days of oral administration of *Cuscuta epithymum* (CE) ethanolic extract. Liver micrographs of male rats treated with saline (a), 50 mg/kg of CE (b), 200 mg/kg of CE (c), and 500 mg/kg of CE (d). Liver micrographs of female rats treated with saline (e), 50mg/kg of CE (f), 200 mg/kg of CE (g), and 500 mg/kg of CE (H). Hepatocytes (yellow arrows), central vein (Star), Kupffer cells (green arrows), hepatocyte apoptosis (red arrows), leukocytes infiltration (green arrowheads), and slight fibrosis around central veins (brown arrows). Hematoxylin and eosin staining, 400X magnification (scale-bar 50µm)

Histological examination of kidney sections of CE-treated animals showed no evident alteration in collecting tubules, glomeruli, or Bowman's capsule (Figure 3). On the other hand, liver pathology revealed that CE treatment mainly at the maximum dose (500 mg/kg) induced slight liver damage. Pathological features, including inflammatory cell infiltration, fibrosis around the central veins, increasing the number of activated Kupffer cells, and hepatocyte degenerations (Figure 4), were observed in this group. 

**Figure 5:**
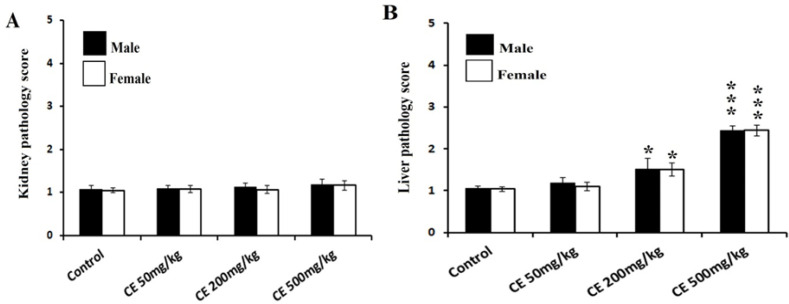
Results of Histopathological scoring of the kidney (A) and liver (B) sections of control and *Cuscuta epithmum* (CE) ethanolic extract-treated rats at doses of 50 (CE50), 200 (CE200), and 500 mg/kg (CE 500). Scoring was done as follows: 1 (normal), 2 (slight damage), 3 (moderate injury), 4 (severe damage) and 5 (very severe damage). Data were analyzed by the Kruskal-Wallis test followed by the Mann-Whitney U test. Differences are represented as *p<0.05 and ***p<0.001 compared to the control group

To make a better comparison, the liver and kidney microscopic slides were scored blindly. Results of the scoring comparison are presented in Figure 5. Histological grading score of kidney sections showed no significant difference between control and CE treated groups. On the other hand, CE at 200 and 500 mg/kg significantly increased liver pathological scores compared to the control scores (p<0.05 and p<0.01, respectively).

## Discussion

In the present study, an essential pharmacological aspect of CE, the plant extract’s toxicological profile, was investigated using *in vitro* and *in vivo* tests.

*In vitro* cytotoxicity assay revealed that CE extract had cytotoxicity against normal (IC_50_ 0.82 mg/kg) and cancer cells (IC_50_ 1.42 mg/kg). Cytotoxicity of CE has been previously studied on cancer cell lines, while data about its effects on normal cells are limited. It is important to discover substances/drugs with selective cytotoxicity for cancer cells (Deniz et al., 2017 ▶). In the study conducted by Jafarian *et al.,* the cytotoxicity of chloroform and ethanolic extracts of CE on breast cancer cells (MDA-MB-468), cervical cancer cells (Hela), and colorectal cancer cells (HT-29) has been investigated. They found that CE ethanolic extract only had a cytotoxic effect on the MDA-MB-468 cell line with an IC_50_ value of 0.34 mg /ml (Jafarian et al., 2014 ▶). Similar results were obtained in a recent study conducted by Firoozan *et al.,* in which the cytotoxicity of methanol, dichloromethane, and N-hexane extracts of CE has been investigated on the mouse (4T1) and human (MDA-MB-231) breast cancer cell lines (Firoozan et al., 2020 ▶). They found that the methanolic extract of CE exhibited IC_50_ values of 0.151 mg/mL for 4T1 and 0.263 mg/ml for MDA-MB-231. It is important to note that the present evidence relies on poor cell selectivity of ethanolic extract of CE.

Acute i.p. administration of the CE extract exhibited mortality at doses greater than 75 mg/kg (i.p. LD_50_ 154.8 mg/kg). Similar results have been reported by Mehrabani and colleagues that investigated the protective activity of CE extract (50-1000 mg/kg, i.p.) in pentylenetetrazol-induced convulsions in mice. They found that CE extract at doses greater than 100 mg/kg caused 26-50% mortality rate in mice (Mehrabani et al., 2007 ▶).

The acute oral toxicity assay results revealed that oral administration of a single dose of CE (175-5000 mg/kg) did not cause mortality, body weight changes or alteration in water and food consumption. According to the Globally Harmonized System of Classification and Labeling of Chemical, substances with acute oral LD_50_ more than 5000 mg/kg should be included in the lowest toxicity class (category No.5) (Winder et al., 2005 ▶). We speculate that this discrepancy between oral LD_50_ (>5000mg/kg) and i.p. LD_50_ (154.8 mg/kg) of CE might be due to differences in its absorption as well as bioavailability. The toxic effect of chemicals/substances can be similar or different between exposure routes indicating the importance of their absorption into the blood (Ning et al., 2015 ▶). Thereby, some substances with low intestinal absorption show much less oral toxicity compared to that induced when they are administered through injection. In oral administration, during the first-pass metabolism in which the liver and gut wall are involved, the swallowed substance's bioavailability is substantially affected (Wang et al., 2015 ▶). 

In the sub-acute study, repeated oral administration of CE extract (50-500 mg/kg) did not alter animals' weight change, food consumption, water intake, or weights of vital organs. The evaluation of organ weights is an integral part of toxicological studies and provides valuable insight into the test substance related-effects (Sellers et al., 2007 ▶).

Interestingly, CE extract at the maximum dose (500 mg/kg) could significantly reduce blood urea level in male and female rats. To our knowledge, no previous research has investigated CE effects on biochemical parameters, including blood urea. However, this result is consistent with what has been found in the study conducted by Koca-Caliskan *et al.,* in which six days of oral administration of methanolic extract of *Cuscuta arvensis* at doses of 125 and 250 mg/kg significantly reduced blood urea nitrogen concentration in the female Sprague–Dawley rats (Koca-Caliskan et al., 2018 ▶).

The most important finding of the present study is the result of liver histopathology. Despite no significant elevation in liver enzymes, noticeable liver damage was observed in the CE-treated rats. Generally, alteration in liver transaminase enzymes may indicate liver damage (Hoshyar et al., 2015 ▶; Ghiravani et al., 2016 ▶). However, evidence shows that normal liver enzymes levels do not always mean that the liver is normal (Lominadze and Kallwitz, 2018 ▶). The results of liver histopathology revealed that CE treatment mainly at the maximum dose resulted in slight degenerative changes and activation of Kupffer cells. Previous studies have shown a direct correlation between the number of activated Kupffer cells and the progression of liver disorders such as liver fibrosis and steatohepatitis (Marra and Lotersztajn, 2013 ▶; Hassanzadeh‐Taheri et al., 2018). Besides, several foci of inflammatory cells were observed in the CE-treated groups (500 mg/kg). Detoxification of xenobiotic plays an essential role in forming these lesions (Hassanzadeh-Taheri et al., 2018a ▶; Hoshyar et al., 2019 ▶). To the best of our knowledge, no study has yet investigated the impact of CE on liver function. In line with our findings, some reports indicated that excessive consumption of dodder (*Cuscuta campestris*) is toxic to cattle and horses (Abutarbush, 2013 ▶). 

We are aware that our study has potential limitations. This study's first and foremost limitation is the lack of hematological evaluation that could be addressed in future research.

We can conclude that CE has *in vitro* and *in vivo* toxicity potentials. CE, mainly at high doses and prolonged consumption, should be considered a causative agent in hepatotoxicity. This herb must be prescribed cautiously by traditional medicine practitioners. 
